# Macroscopic properties of buyer–seller networks in online marketplaces

**DOI:** 10.1093/pnasnexus/pgac201

**Published:** 2022-10-06

**Authors:** Alberto Bracci, Jörn Boehnke, Abeer ElBahrawy, Nicola Perra, Alexander Teytelboym, Andrea Baronchelli

**Affiliations:** Department of Mathematics, City, University of London, London EC1V 0HB, UK; Graduate School of Management, University of California Davis, 1 Shields Ave Davis, CA 95616, USA; Chainalysis Inc, New York, NY 10011, USA; School of Mathematical Sciences, Queen Mary University of London, London E1 4NS, UK; Department of Economics, University of Oxford, Oxford OX1 3UQ, UK; Department of Mathematics, City, University of London, London EC1V 0HB, UK; UCL Centre for Blockchain Technologies, University College London, London WC1E 6BT, UK; The Alan Turing Institute, London NW1 2DB, UK

**Keywords:** online marketplaces, complex networks, buyer–seller networks, dark web marketplaces

## Abstract

Online marketplaces are the main engines of legal and illegal e-commerce, yet their empirical properties are poorly understood due to the absence of large-scale data. We analyze two comprehensive datasets containing 245M transactions (16B USD) that took place on online marketplaces between 2010 and 2021, covering 28 dark web marketplaces, i.e. unregulated markets whose main currency is Bitcoin, and 144 product markets of one popular regulated e-commerce platform. We show that transactions in online marketplaces exhibit strikingly similar patterns despite significant differences in language, lifetimes, products, regulation, and technology. Specifically, we find remarkable regularities in the distributions of transaction amounts, number of transactions, interevent times, and time between first and last transactions. We show that buyer behavior is affected by the memory of past interactions and use this insight to propose a model of network formation reproducing our main empirical observations. Our findings have implications for understanding market power on online marketplaces as well as intermarketplace competition, and provide empirical foundation for theoretical economic models of online marketplaces.

Significance Statement Online marketplaces have become very successful in the past 20 y, with dark web marketplaces (DWMs) also proliferating on the dark web to satisfy the demand for illicit goods. However, the networks that result from interactions between buyers and sellers on these platforms are poorly understood. Here, we investigate these networks by analyzing 245M transactions on one e-commerce platform and 28 DWMs. Despite many differences between the marketplaces, we find remarkable regularities in user behavior and propose a simple model reproducing the main empirical observations. The results shed light on buyer–seller networks and their formation mechanisms, highlighting the central role of buyer memory and preferential attachment mechanism, and have important implications for the understanding, design, and regulation of these platforms.

## Introduction

Much of online trade happens on regulated and unregulated online marketplaces. Regulated online marketplaces include Amazon, Craigslist, eBay, Walmart, Alibaba (China), Rakuten (Japan), Gumtree (UK), and Mercado Libre (South America). Unregulated online marketplaces, such as Silk Road, AlphaBay, and Hydra, which specialize in the sale of illicit goods, have proliferated on (and disappeared from) the dark web since the introduction of Bitcoin (1–4). The amount of transactions in online marketplaces is vast and growing. For example, in 2020 Amazon reported a net revenue of 386B USD (5), while in 2019 the ecosystem of dark web marketplaces (DWMs) had reached a total volume of 4B USD (2).

Online marketplaces are commercial websites that allow participating buyers and sellers to exchange information about prices and products and to execute transactions  (6–8). Sellers can usually post an ad for their product that includes a product description, a price and a shipping method. Buyers instead can see all relevant product ads matching search keywords, and have access to reviews and seller ratings. When a purchase is made, the payment is processed through the platform, while shipping is usually taken care of by the seller.

Despite the importance of online marketplaces for e-commerce and global trade (9,10), little is known about their empirical properties, transaction patterns and the resulting buyer–seller networks. The properties of the transaction network could, however, provide important insights into the presence of market power (11,12), the nature of interplatform competition (13,14), product design (15), the effects of reputation on sellers’ revenue growth (16), and the long-run sustainability of the platforms (17). Moreover, measuring properties of the buyer–seller networks could help provide empirical foundations for theoretical models of online marketplaces, from the estimation of model parameters to suggesting specific model mechanisms. However, buyer–seller networks in online marketplaces have specific features that make them different from other networks (e.g. social networks): they exhibit a naturally bipartite structure; most transactions (links) occur between anonymous agents; transaction activity might be infrequent and sporadic. Moreover, the structure of buyer–seller networks could depend on the nature of the traded products, on the types of buyers and sellers, on the user experience on the marketplace, or even on the legal, institutional and geographic constraints.

One strand of prior work relevant to our paper has touched on various aspects of regulated online marketplaces. For example, the role of reputation and feedback (18–20) has been identified as one of the main drivers of the worldwide success of regulated online platforms (21). Other work has looked at consumer search and the effect of rankings on product choice (22–25), online auction markets (26–29), market microstructure (30, 31), and price formation in online markets (32–36). [For a more complete but less up-to-date review, see ref. (6).] Another strand of research has studied unregulated marketplaces. This work has focused on country-specific studies (37–39), the effects of closures and law enforcement raids (2,3,40–42), the characterization of the trade of specific goods (43–45), the importance of geography (46,47), or sociological interview-based studies (39, 48). However, most work on unregulated online marketplaces was limited to specific markets, and focused on information available from public listings (e.g. using crawled data) (1, 40,43–46).

In this paper, we focus on patterns in transactions, which typically cannot be publicly observed either on regulated or unregulated online marketplaces. We analyze two datasets. The first dataset contains 220M transactions between 99M buyers and 7.4M sellers, which occurred in 144 randomly sampled product markets of one regulated e-commerce platform between 2010 and 2020, for a total volume of over 10B USD. The second dataset contains 25M transactions involving 17M entities with a total volume of 4.2B USD, which occurred in 28 major DWMs between 2011 and 2021, for a total volume of 4.2B USD (for more details on the datasets see the “Materials and methods” Section). In both cases, the datasets cover all transactions, which occurred in each corresponding market.

We observe striking similarities in user behavior across online marketplaces, despite their significant differences. First, we find that the number of transactions, amount, interevent time and time between first and last transaction are highly heterogeneous across users but follow consistent fat-tailed distributions across all marketplaces. Then, we show that individual behavior is influenced by past purchases similarly (albeit less strongly) to what is observed in the renewal of past ties in social networks (49,50). Finally, we propose a simple model of buyer–seller interactions that reproduces the main stylized facts of the data and emphasizes the critical role of preferential attachment (51, 52) and memory in the market dynamics.

## Results

### Empirical properties of buyer–seller networks

**Fig. 1.**
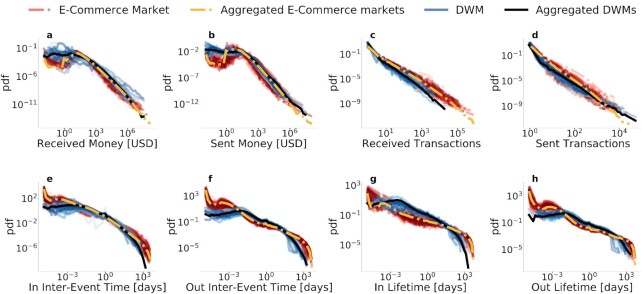
Online marketplaces show strikingly similar patterns according to different aggregated quantities. Top (a to d): distributions of 4 analyzed users aggregate quantities: money and number of transactions both sent and received. Bottom (e to h): distributions of four analyzed users temporal quantities: the interevent time (time between successive transactions) and the lifetime (time between first and last transaction) both measured in days. Each blue line represents one DWM, the black line is the distribution built aggregating all DWMs together, each dashed red line represents one e-commerce platform market, while the dashed yellow line is the distribution aggregating all e-commerce markets. Similar patterns are observed between different markets in the same platform, but also across regulated and unregulated online marketplaces.

In order to characterize the buyer–seller networks, we start by analyzing different aggregate user-level quantities. First, we study the distributions (for each market) for the number and amount of user transactions. Results for all DWMs and for each e-commerce market are shown in Figs. 1(a) to (d), where black and yellow lines are obtained by aggregating all users in the respective datasets. Single distributions display common behavior, spanning several orders of magnitudes. It is important to note that distributions are computed without any rescaling or manipulation of the data, and that higher values generally reached by the regulated platform in all distributions are exclusively due to the different platform sizes. The slight discrepancy between the distributions in the total number of received transactions can be ascribed to the different nature of the two datasets: While in the DWM dataset sellers can withdraw the earnings from several market trades at once, in the e-commerce data each transaction corresponds to a single purchase.

We then analyze the temporal dimension of the data. We focus on the distribution of user lifetimes, defined as the time between the first and last user transaction in the market, and the interevent time between two successive transactions of the same user. Again, we find remarkable regularities across different DWMs and different regulated product markets, as shown in Figs. 1(e) to (h). In these distributions, as before, we also observe the effects of different sizes of marketplaces. The similarity between different distributions is particularly pronounced in the meaningful timescales between an hour and a month/year. Discrepancies for longer periods are due to the different lifetimes of the markets, whereas discrepancies for shorter timescales can be explained by the different nature of the two datasets: precise timestamps on transaction data for the regulated marketplaces vs. times at which the transaction is actually added to the Bitcoin blockchain (which depends on its algorithm) for the DWM dataset.

Having considered buyers and sellers separately, we now investigate the dynamics of buyer–seller relationships and the evolution of the buyer–seller network. We limit this analysis to e-commerce markets, since DWMs data do not contain buyer–seller links (see the “Materials and methods” Section for more details). We first consider how single users distribute their purchases across sellers: for example, buyers could purchase equally from multiple sellers or, alternatively, buyers could show loyalty to one seller from which they do most of their purchases. A standard way to quantify how distributed or concentrated this pattern is to compute the normalized entropy for the purchases of each buyer *i* as in Eq. 1, and then compute its distribution for all markets. The normalized entropy is defined as

**Fig. 2.**
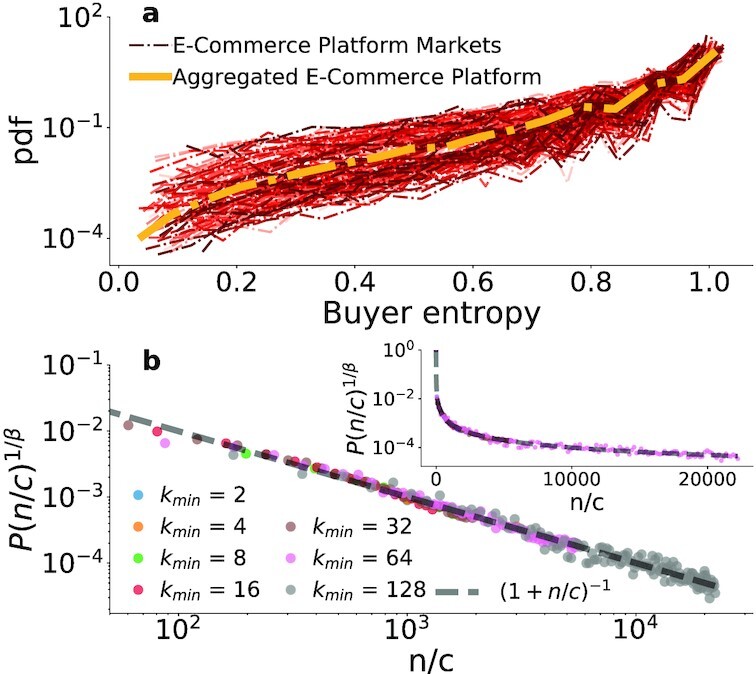
Buyer memory affects their purchase decisions. (a) Normalized buyer entropy distribution for each e-commerce market (red), and the whole e-commerce platform (yellow), excluding users with zero entropy (mostly users with one transaction, see Fig. S1 in the Supplementary Material) for visual clarity. The fat-tailed distributions span the full [0,1] range, with most buyers almost equally buying from multiple sellers. (b) *P*(*n*) is the probability to buy from a new seller after a buyer has already bought from *n* different ones. Each degree class *k_min_* ≤ *k* ≤ 2 *k_min_* − 1 is rescaled according to the fitted value of *c* and β (see Table S1 in the Supplementary Material for the values), with Eq. 2 (dashed line) well reproducing the memory effect on the buyers’ behavior: the more sellers they try, the less likely they are to buy from a new one.

(1) \begin{equation*} e_i = - \sum _{j=1}^{J}n_i^j \log _2(n_i^j) / \log _2(J) , \end{equation*}

where $n_i^j$ is the share of buyer *i*’s purchases from seller *j* and we sum over the *J* sellers the buyer purchased from. Fig. 2(a) shows that the distributions, computed for each market, are fat-tailed, with buyers populating the full [0,1] support but with most of the mass toward the top, meaning that most buyers buy a similar number of times from the different sellers they purchase from. Buyers with zero entropy, who buy from just one seller, were excluded from the figure for visual clarity, but these were almost exclusively buyers who only made a single purchase (see Fig. S1 in the Supplementary Material). In Fig. S2 in the Supplementary Material, we further compare the distributions against a null model obtained reshuffling the transactions in the dataset (preserving buyers activity and sellers attractiveness), where we find lower heterogeneity and higher tendency toward high values of entropy. This implies that the empirical entropy distributions show a broader non trivial range of behaviors interpolating between perfect exploration and exploitation.

The observed normalized entropy distributions are compatible with different kinds of temporal patterns produced by two possible choices: either buyers choose to engage with new sellers they have never purchased from (i.e. exploration) or they return to a known seller (i.e. exploitation). We investigate these dynamics by leveraging insight from the social networks literature, where several studies have investigated how users explore and exploit social connections by renewing previously activated ties or by establishing new ones (49,50). Indeed, across different types of social networks, the temporal evolution of links that a person forms with their contacts can be captured by the following expression:

(2) \begin{equation*} P(n) = \left(1+n/c(k_{min})\right)^{-\beta (k_{min})} , \end{equation*}

where— now using the language of online marketplaces —*P*(*n*) is the probability that a buyer (node) of degree *n* (who has already bought from *n* different sellers) chooses to buy from a new seller, while *c* and β are positive constants, depending on the final degree of the buyer, which measure their propensity to explore new sellers and thus the effect of memory. Following the procedure proposed in (49) (see Supplementary Material for more details), we group nodes in different classes according to the final degree: a buyer is in class *k_min_* if the final degree *k* satisfies *k_min_* ≤ *k* ≤ 2 *k_min_* − 1, starting from *k_min_* = 2. For this computation, we aggregate all markets together in order to have a representative sample in classes with higher *k_min_*. If a user is present in multiple markets, we keep its activity in different markets separated (i.e. effectively considering her as different users). We then fit Eq. 2 to each node class obtaining a value of *c*(*k_min_*) and β(*k_min_*) (see Table S1 in the Supplementary Material).

Results are shown in Fig. 2(b). Since different classes feature different values of β and *c*, we plot a rescaled *P*(*x*)^1/β^ as a function of *n*/*c*. Indeed, Eq. 2 becomes 1/(1 + *x*) (dashed line in Fig. 2b) for every degree class *k_min_*, where *x* = *n*/*c*. In other words, we re-scale both axes assuming the empirical behavior is captured by Eq. 2. As shown in Table S1 in the Supplementary Material, the parameter values are independent of the degree class and suggest a weaker (β ∼ 10^−1^) effect than previously observed in social networks (0.48 ≤ β ≤ 2) (50). The close fit of the data to the predicted memory for different *k_min_* indicates the applicability of Eq. 2 in the dynamics of buyer–seller relationships. While users have different propensities to explore new sellers, they follow the same mechanism: the more sellers a user has bought from, the less likely is their next purchase from a new seller.

### Modeling buyer–seller networks

In order to understand possible mechanisms that drive the properties of buyer–seller networks, we propose an agent-based model aimed at capturing the patterns observed in the previous section. The main features of the model are:

1. *Activity*. The rate at which buyers make transactions. As shown in Fig. 1, in both e-commerce and DWMs buyers feature heterogeneous propensities to make purchases.
2. *Memory*. When making new transactions buyers can either choose a seller they already bought from or pick a new one. As shown in Fig. 2(b) and discussed above, buyers have a memory of the sellers they had interacted with, and this memory affects their future purchases.
3. *Preferential attachment*. The attractiveness (i.e. popularity) of a seller is proportional to the number of their sales. This attractiveness captures the fact that, in online marketplaces, sellers are rated based on the feedback they receive from the buyers (i.e. customer reviews), and buyers prefer sellers with higher ratings, other things equal (18,19,53–55). Here, we focus on the number of sales rather than sale volume to capture the fact that it is mainly frequency of transactions that matters for seller reputation.

Given these three ingredients the model dynamics is as follows. The system consists of *N* buyers and *M* sellers. At *t* = 0 we assign the activity *a_i_* to each buyer *i*. Each seller *j* starts with attractiveness *A_j_* = 1. At each time step *t*, each buyer makes a purchase with probability *a_i_* · Δ_*t*_, where Δ_*t*_ is the simulation time step (fixed to 1 from now on). A buyer who interacted with *n* sellers in the past has probability *P*(*n*) = (1 + *n*/*c*))^−β^ of choosing a new seller and 1 − *P*(*n*) of returning to a known one. In the first case, the buyer selects a new seller *j* proportionally to their attractiveness (56) *A_j_*, in the latter, the buyer selects it proportionally to the number of previous interactions. In other words, buyers select sellers either according to past purchases or to their popularity. In both cases the attractiveness of the seller is increased by μ. This model produces a bipartite temporal network: at each time step *t* we build a network in which two types of nodes— buyers and sellers—are linked if the buyer has purchased from that seller at time *t*. These networks are then combined together in an aggregated network, where each buyer–seller link is weighed according to the number of purchases between that buyer and that seller across time.

Compared to other activity-driven models developed to capture the temporal evolution of different social networks (49,50, 57), our model extends the framework to bipartite networks of buyers and sellers and introduces the preferential attachment guiding the buyer selection process. Henceforth, we will refer to the model lacking preferential attachment, proposed in (49), as *Model NoPA*. We will also consider a version of the model that does not include the memory element (*Model NoMem*). Comparing these versions of the model will allow us to identify the role played by the different mechanisms.

**Fig. 3.**
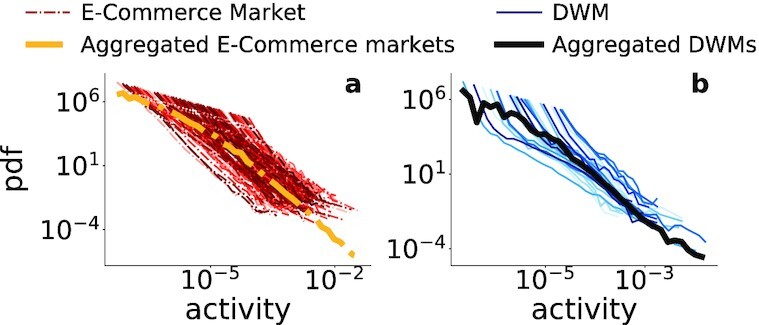
Empirical activity distributions. (a) Activity distribution for all e-commerce markets in red, and activity distribution of all aggregated markets in yellow. (b) Activity distribution for all DWMs in blue, and activity distribution of all aggregated DWMs in black.

A standard way to define and measure user activity in a (social) network is *a_i_* = *n_i_*/Σ_ℓ_*n*_ℓ_, where *n_i_* is the number of purchases made by buyer *i*, where the sum is over all buyers in their market. In Fig. 3, we show the activity distributions of all e-commerce markets (a) and all DWMs (b). While curves exhibit fat-tailed behavior, they no longer overlap due to different activity ranges and shapes in different product markets. As a result, we need to use market-specific empirical activity distributions as inputs for our model.

We now fit the model to the e-commerce data. As mentioned above, since the DWM dataset does not contain the full bipartite buyer–seller network, we cannot test all the model predictions on the DWM data. We employ a data-driven approach, fine-tuning the model to each single market so we can more faithfully compare the simulation results with the empirical buyer–seller networks. In the main text, we show results for two different product markets, 26 more are shown in Figs S3 to S6 in the Supplementary Material, for a total of 28 markets (see Supplementary Material for the sampling procedure). We fix parameters β = 0.1 and *c* = 0.001, which we fitted previously (see Table S1 in the Supplementary Material), and use the empirical activity distributions as measured in the data (see Fig. 3a) to reflect the observed heterogeneity across different markets. The value of μ is determined with Maximum Likelihood Estimation for each market (see Supplementary Material for more details, and Table S3 for the fitted values).

**Fig. 4.**
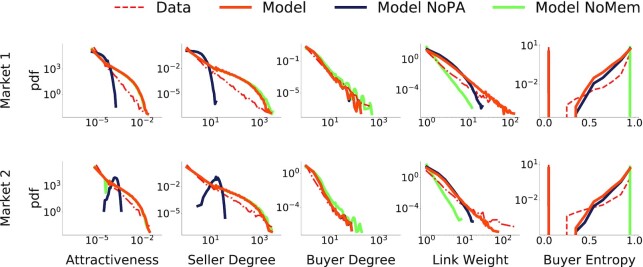
The model reproduces different properties of buyer–seller networks. Each row corresponds to a different market (see Figs. S3 to S6 in the Supplementary Material for other markets), whose simulations parameters are individually calibrated as detailed in the main text. From left to right, we show distributions for different quantities: attractiveness, seller degree, buyer degree, link weight and seller entropy. The comparison with the two model variations, without preferential attachment or without memory, shows the key role of both parameters in shaping the network: preferential attachment is crucial in reproducing highly active sellers, whereas buyer memory is fundamental to capture the heterogeneity of buyer–seller relationships.

Results are in Fig. 4. We first compare the model’s output with the empirical distributions of the final seller attractiveness and degree. The attractiveness of a seller *j* is their market share *A_j_* = *s_j_*/Σ_ℓ_*s*_ℓ_, where *s_j_* is the total number of sales of seller *j* and the sum is over all the sellers. Fig. 4 shows that the model reproduces both distributions well, while the *NoPA* variation of our main model (without preferential attachment) fails to capture the heterogeneity (up to six orders of magnitude) of these curves, emphasizing how preferential attachment is key to reproducing the presence of very active sellers. We then consider the buyer side of the network. We first study the degree distribution. Fig. 4 shows that the model captures the empirical distributions, while the absence of buyer memory generally leads to a small overestimation of the tails, since it does not induce the repetition of past interactions with a subset of buyers.

**Fig. 5.**
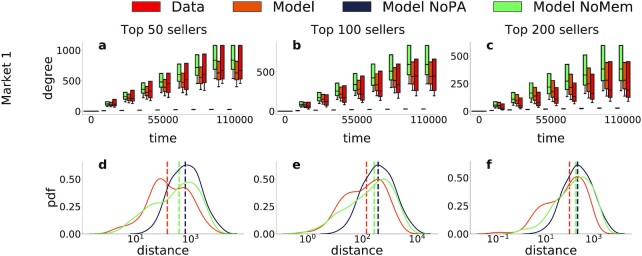
Model reproduces the temporal evolution of the top sellers degree distribution. Top (a to c): Temporal evolution of the degree distribution of the top 50 (a), 100 (b), and 200(c) sellers, representing the distribution at nine equally spaced time steps with boxplots ranging from the first to the third quartiles, whiskers extending from 2.5th to 97.5th percentiles. Results are shown for one product market, all other markets are shown in Figs. S7 to S10 in the Supplementary Material. Bottom (d to f): Distribution of the distance between the empirical and model(s) median degree of the top 50(left), 100(center), and 200(right) sellers, for all product markets and time steps, and the three considered models. Vertical lines represent the distributions median, showing that the model median is always smaller than the alternatives. The model better captures the temporal evolution of the top sellers degree for all product markets than the alternatives neglecting either the preferential attachment or the memory mechanism.

Thus far, we have considered node-level properties aggregating detailed information on the links. For example, the attractiveness only accounted for the total number of links, whereas the degree only captures the total number of different buyers or sellers that the user has interacted with. To better understand how the model performs in reproducing finer details of the buyer–seller network structure, we test our model against two other properties of the aggregated network: link weight—the number of transactions between a buyer and a seller—and the buyer entropy, as defined in Eq. 1. Our main model outperforms its two variations in reproducing the shape and tails of the link weight distribution. In particular, the memory mechanism appears to be fundamental in reproducing repeated transactions between a buyer and a seller. The buyer entropy distribution is again well-captured by the model and shows how the memory mechanism is key to capturing the diversity of relationships buyers establish with different sellers. Indeed, the *NoMem* model produces only entropy values close to 0 and 1; this happens because without memory, a buyer almost never finds any previous seller, hence buyers making more than one purchase almost always buy from new sellers.

We have seen that our model is able to capture various aspects of the final aggregated buyer–seller network. The next step is to see whether our model can also reproduce the temporal evolution of the buyer–seller network. To investigate this, we focus on the degree of top sellers since we previously showed these sellers generate the largest activity and volume on these markets. We measure time by the total number of purchases made. Results are shown in Figs. 5(a) to (c), where we plot the temporal evolution of the top 50 (a), 100 (b), and 200 (c) seller degree distribution for one illustrative product market. In doing so, we compare the model to its two variations and the data. Results for more product markets are shown in Figs. S7 to S10 in the Supplementary Material. The main model is able to reproduce the temporal evolution of the distributions, as clearly shown by the cores (i.e. interquartile ranges) overlapping at different times. We further compute the absolute value of the difference between the mean of the models’ distribution and the mean of the data, for each of the nine equally spaced time steps and for all 28 simulated product markets. As shown in Figs. 5(d) to (f), the model is better able to reproduce the temporal dynamics for all simulated markets. Indeed, the median of the distance distributions is always smaller in the main model than the two other model variations.

## Discussion

In this paper, we have analyzed 244M (25B USD) transactions occurring on regulated and unregulated online marketplaces. First, we have revealed remarkable regularities in the aggregate static and temporal properties of the buyer–seller networks, both for buyers and sellers. Then, we have revealed how buyers are affected by the memory of past interactions. Finally, we have proposed a model, which captures the main stylized facts of the data, based only on three well-known network formation mechanisms of online marketplaces: buyers have different propensity to make purchases, they remember the sellers they purchased from, and they are more likely to buy from successful sellers.

It is important to highlight the limitations of our study, which also represent directions for further work. First, while our study is based on (pre-processed) blockchain data, access to DWM server logs could provide more detailed information on some specific markets, for instance, the directed buyer–seller links, which are not observable in our data. Second, the model could be further enriched with other known mechanisms: pricing dynamics (58, 59), product search ranking (22–25), variable (e.g. also negative) customer reviews (20), sellers entering or leaving the platform (60), and recommendation algorithms (61). Finally, including richer economic incentives (e.g. strategic behavior) to model buyers’ and sellers’ decisions could shed light on how agents could exploit their market power. In particular, the inclusion of strategic behavior would also to drop phenomenological rules such as preferential attachment, which would naturally result from the agents’ behavior (62,63). A deeper understanding of economic incentives and equilibrium behavior in buyer–seller networks could ultimately inform market design and regulation of online marketplaces.

Nevertheless, our work supports and extends previous findings. The fat-tailed heterogeneous curves in Figs. 1(a) to (d) substantiate previous observations of high concentration in DWMs: wholesale (47), few sellers (40), or few buyers (46) were found responsible for the largest part of volumes in smaller samples of data. The fat-tailed interevent time distributions, spanning times between a second and a year, are compatible with the bursty nature of several social activities (64,65), and the finding about a shared memory kernel further points to a similarity between social and economic activities (49,50). Taken together, our results could inform and enrich economic models where heterogeneity assumptions are now commonplace (14) but empirical evidence on the structure of buyer–seller networks has not yet been introduced.

The regularities observed in Fig. 1 are surprising given the differences in the marketplaces covered by our data: transactions on the clear web with state enforcement of contracts (66) vs. transactions on the dark web that rely mainly on reputation and self-governance (54); the sale of only regulated products vs. mainly unregulated products; and the use of fiat vs. the use of cryptocurrencies. And, indeed, there is both substantial heterogeneity in product markets in the e-commerce dataset and several differences across marketplaces in the DWM dataset (e.g. existence time period, geography, product focus, etc.). Our model suggests specific mechanisms that drive the regularities across the two datasets. Sellers build a reputation that makes them more attractive to buyers who, in turn, are affected by their memory of the sellers they already purchased from. In particular, the presence of both memory and preferential attachment is fundamental in reproducing both local and global properties of the buyer–seller network, as already shown for the intrinsically different social networks (49,51,52,64). However, commercial interactions exhibit important differences compared to social interactions, with preferential attachment playing a dominant role in the market dynamics.

Our results point toward alternative strategies to attempt to reduce trading of illicit goods on DWMs. Historically, DWMs have been closed after long and expensive operations targeting the market admins in order to arrest them and shut down the servers (67). However, the high degree of concentration, the importance of preferential attachment, and the memory kernel in the buyer dynamics, all suggest that limited observations of the market dynamics could give a clear enough picture of who the key actors of these networks are. For instance, key sellers will most likely attract most of the observed purchases from the more active buyers, and stopping them would effectively stop a large part of the market trade. In this regard, our model could also be used to produce candidate synthetic DWM buyer–seller networks to quantitatively study and simulate the effects of targeting “key players” on marketplaces (68).

Finally, a better understanding of buyer–seller network formation could have consequences for market design and regulation. For example, fat-tailed distributions show a high degree of concentration on both buyer and seller sides of the marketplaces: just a few agents (both on the buyers and seller sides) are responsible for a vast majority of the transaction volume. While buyer market power appeared in analyses of labor monopsony and retailers (69,70), our empirical finding of buyer concentration calls for a deeper understanding of buyer power in online marketplaces. Moreover, these observations can also inform theoretical economic models of online marketplaces, providing empirical backing to heterogeneity assumptions and suggesting specific values for parameters or shapes for distributions. Also, we find signs of both local (memory) and global (reputation) mechanisms in the structure and evolution of buyer–seller relationships. Thus, the inclusion of memory and reputation in previously developed models can improve our understanding of the pricing of network effects (12), interplatform competition (14) and long-run sustainability of the platforms (17).

## Materials and methods

### DWMs

DWMs are illegal unregulated commercial websites. They operate similarly to other online marketplaces, such as Gumtree or Craigslist. To improve anonymity DWMs are reached through browsers supporting the onion protocol, and use cryptocurrencies, mainly Bitcoin, as the main currency. While all Bitcoin transactions are publicly available, they record money exchanges between addresses, and a user can generate a new address (identifier) at each transaction to favor anonymity. As a result, the data need to be preprocessed to cluster addresses into individual entities in order to perform any economic analysis. Our dataset has been preprocessed by Chainalysis Inc. (71) to map addresses into entities (see Supplementary Material for more details).

Our dataset contains the entire transaction data of 28 entities corresponding to DWMs between 2011 June, and 2021 February (see Fig. S11 and Table S2 in the Supplementary Material). These markets all have an average daily volume of more than 15,000 USD, in order to be able to reliably measure different observables, and include all relevant DWMs as identified by law enforcement agencies (67). The data contain all transactions received or sent by DWMs, excluding services such as exchanges (Bitcoin trading exchanges allow users to trade Bitcoin). Note that the data hide the direct buyer–seller link, because the money pass through the platform during the transaction.

### E-commerce platform

E-commerce platforms are regulated online marketplaces where sellers can post ads for products. Buyers and sellers can generally be both individuals or businesses. The payment is usually processed by the platform, but the shipping is handled by the seller. Sellers receive feedback from buyers, which together with product categorization helps people navigate the platform and choose what to buy.

The data used in this study consist in all the purchases made on 144 product markets from a popular e-commerce platform since 2010. The 144 product markets have been randomly selected from the markets that were active during the entire time period. The data cover only one geographical region. Similarly to the DWM data, the transaction data include: timestamp of the transaction, pseudonyms for buyer and seller, and the amount spent in the transaction. One key difference is that the data show the direct link between buyer and seller, forming a bipartite buyer–seller network and allowing for a more fine-grained analysis. For more details on the data see Fig. S12 in the Supplementary Material.

### Model simulation

Each simulation is tuned to simulate one specific product market. We fix the agents population according to the data: number of sellers *N*, number of buyers *M*, and simulation total number of time steps *T*, to fix the average total number of transactions in our simulations as in the data: 〈*a_i_* · Δ*t* · *T* · *N*〉 = *t*, where *a_i_* is the buyer activity as defined in the main text, Δ*t* is the simulation time step (fixed to 1) and *t* is the total number of transactions present in the data. We realize 30 different realizations for each parameter set, and aggregate the final results.

## Preprints

A preprint of this article is published at

https://arxiv.org/abs/2112.09065.

## Supplementary Material

pgac201_Supplemental_File

## Data Availability

Synthetic data reproducing the main properties of the original dataset are available in the public OSF repository dataset for the paper *”Macroscopic properties of buyer–seller networks in online marketplaces”* at https://osf.io/rw2ce/
