# Supplementary material for: Macroscopic properties of buyer–seller networks in online marketplaces
Source: PNAS Nexus. 2022 Oct 6;1(4):pgac201. doi: 10.1093/pnasnexus/pgac201 (PMC9802486; doi:10.1093/pnasnexus/pgac201)
Supplement: pgac201_Supplemental_File [file pgac201_supplemental_file.pdf]

PNAS nexus

Supplementary Materials for

Macroscopic properties of buyer-seller networks in online marketplaces

Alberto Bracci, Jörn Boehnke, Abeer ElBahrawy, Nicola Perra, Alexander Teytelboym, and Andrea Baronchelli*

* Corresponding author. Email: a.baronchelli@turing.ac.uk

This PDF file includes:

Supplementary Text
Tables S1 to S3
Figures S1 to S11
References *(1-12)*

Supplementary Text

Dark Web Marketplaces

Dark Web Marketplaces (DWMs) are unregulated commercial websites. They operate similarly to other online marketplaces, such as Gumtree or Craigslist. In most cases, the buyer sends money to the DWM which keeps the money until the client has confirmed receipt of the goods, upon which the funds are released to the seller after taking a small fee. Customers may also leave reviews, contributing to the sellers reputation. To favour anonymity DWMs are reached through browsers supporting the onion protocol [1], to find their updated domain one can navigate one of the many specialised websites like DeepDotWeb and darknetlive [2]. DWMs use cryptocurrencies, mainly Bitcoin, as the main currency.

Our dataset contains the entire transaction data of 28 dark marketplaces between June, 2011, and July 2020. These markets all have an average daily volume larger than 15,000 USD, in order to be able to reliably measure different observables, and include all relevant DWMs as identified by law enforcement agencies. Each marketplace can be represented as an egocentric network around the DWM, a star where the marketplace is the central node and its nearest neighbours represent marketplace users. A directed edge represents a transaction occurring between the DWM and one of its nearest neighbours. Note that the data hide the buyer-seller direct link, because the money go through the platform during the purchase.

All transactions ever done in Bitcoin are publicly available, and can be downloaded by installing Bitcoin core software or through various third party APIs like Blockchain.com. Each transaction is recorded through its time, exchanged amount, source and destination addresses. An address is an alphanumeric identifier, and a user can generate a new one every time he does a transaction, and this what now generally happens for anonymity reasons. For this reason, the data need to be pre-processed to cluster addresses into individual entities in order to perform any economic analysis.

Our dataset has been pre-processed by Chainalysis Inc following approaches as in [3, 4, 5]. This process uses established heuristics [6, 7, 8, 9, 10] in order to map addresses into entities. This process is unsupervised, as there is no ground truth data to rely on. Avoiding false positives in the clustering is crucial, as this data have been used in many law enforcement investigations related to illicit activities [11]. This means that if an addresses does not meet the heuristics conditions it is left unclustered, and therefore not all addresses linked to DWMs or their admins may be correctly included in the market entity.

Chainalysis Inc. also identifies the clustered entities corresponding to a number of dark web marketplaces. Our dataset contains their full transaction history, meaning all the transaction in which the markets either send or receive money from other entities. Bitcoin trading exchanges were excluded from the list of trading entities, since we focus on the users' direct interaction with the dark marketplace. (Bitcoin trading exchanges are platforms that allow users to trade Bitcoin for other cryptocurrencies or fiat currencies.)

We collect additional information on the analysed marketplaces from different sources, including the Gwern archive [12], law enforcement agencies reports and dedicated online forums. We focused our attention on the creation and closure dates of these markets, in order to correctly interpret the transaction data. We report the lifetimes of the selected markets in Figure S11, color coding by the daily average number of transactions as proxy for the market size. For more details on the markets, see also Table S2.

Computing the Memory Kernel

In this section we describe the computations made in order to estimate the buyer memory parameters c,β. As detailed in the main text, buyers are grouped in different classes according to their final degree (number of different sellers they purchased from) at the end of the considered periods. Classes are divided in powers of 2, for example the first class includes buyers with degree 1, the second with degree between 2 and 3, the third between 4 and 7 and so on. In our dataset, a buyer has a unique identifier in each market product, not allowing to follow their behavior across different markets. In order to reduce the noise in the data, all markets are aggregated together for the following computations.

In order to estimate the memory parameters from eq.2, defined in the main text, the first step is to estimate the conditional probabilities $P_k(n+1|n) = P_k(n)$ of buying from a new $n+1^{th}$ seller when you already bought from n different ones. To do so, we count the number of buyers $b_k(n)$ in class k who go from degree n to $n+1$, and we count the total number of purchases $p_k(n)$ they made when they had degree n.

$$P_k(n) = b_k(n)/p_k(n) \tag{1}$$

In order to reduce the noise on the computation of $P_k(n)$, we limit the computation to $n \leq k$. This way, all buyers in degree class k go from degree n to $n+1$, as their final degree is at least equal to k. The numerator is therefore constant, and equal to N_k, the number of buyers in degree class k. Equation $P(n)$ then reads:

$$P_k(n) = N_k/p_k(n) \tag{2}$$

Assuming that for a given degree n events are independent, or in other words that users behave independently of each other, and checking that $1 << N_k << e_k(n)$, we can estimate the uncertainty of $P_k(n)$ as follows:

$$\sigma(P_k(n)) = \sigma_k(n) = \sqrt{P_k(n)(1-P_k(n))/e_k(n)} \tag{3}$$

Having estimated the curve $P_k(n)$ for each degree class k, we can fit eq.2 to each curve separately. To do so, we do a numerical least square optimization, estimating the values of β and c for each degree class. Results are shown in Table S1.

Sampling of Product Markets

The e-commerce platform contains data on 144 product markets. We sample 28 DWMs to fit with our model. The 28 product markets are sampled to ensure all products are represented. In particular, products can be grouped together in higher-level markets, from which we sample on product each. To make an example: our dataset may contain two product markets in the fruit group, namely apples and pears. In the sample for the model simulation we choose only one of the two, taking care that the 28 sampled product markets are representative of the heterogeneous market size of our dataset.

Model parameters estimation

As detailed in the main text, we employ a data-driven approach to estimate the model parameters for each product market. The model parameters, described in the main text, are chosen in the following way. The number of time steps of the simulation T is chosen such that the average number of transactions in the simulation is equal to that of the data. Similarly, the number of buyers and the number of sellers are the same as in the market we are simulating. The users' activity is instead drawn from the empirical activity distribution. The memory parameters, namely c and β, are fixed to 10^{-3} and 10^{-1} respectively, values that well represent the different values fitted from the empirical dataset as shown in Table S1. Finally, the preferential attachment parameter μ, describing the increment of a seller's attractiveness after a sale, is estimated by Maximum Likelihood Estimation (MLE). To do so, we simulate the model for each value of μ on a grid, ranging from 1 to 500, and then compute the associate negative log-likelihood computed comparing the data to the simulated attractiveness distribution, and choose the value minimizing the quantity. For instance, the likelihood of our empirical data is computed as the product of the probabilities that each data point had according to the attractiveness distribution built with the simulated data. We employ this simple approach to estimate μ, by only analysing the attractiveness distribution, as the scope of this work is to study and reproduce stylized facts, and not to propose a detailed model precisely reproducing all details of a given product market. For this reason, even the value of μ itself assumes relative importance, as its order of magnitude determines the agreement with the data, but small variations in the precise value are meaningless in the context of this study. For completeness, in Table S3 we show the fitted value of μ for each product market.

Supplementary Figures

In Figure S1, we show the histogram of the percentage of users with entropy zero doing just one transaction, in each product market. This percentage is always greater than 75%, but actually over 90% in most cases, showing how buyers with entropy zero can effectively be neglected when showing the buyer entropy distribution.

In Figure S2, we test the entropy distribution from Figure 2a of the main article against a null model. In Figure S2a, we reproduce the buyer entropy distribution for each market (in red) and all markets (in yellow) for the e-commerce dataset. In Figure S2b, we show the same distributions for the same dataset reshuffling the transactions link, such that buyers maintain the same number of transactions but with randomly chosen sellers. The latter distributions show a narrower support with high values of entropy, showing that memory effects disappear in a null model preserving just the activity of buyers and sellers.

In Figure S3 to S6 we show results of model simulation for 26 other product markets. The results show how the model is able to capture the main stylised facts of the buyer-seller network structure, with memory and preferential attachment both necessary to capture different aspects of the structure.

In Figure S7 to S10 we show the temporal evolution of the top 50, 100 and 200 sellers degree distribution for 26 other product markets, represented as boxplot for 9 equally spaced time steps. The model is consistently able to reproduce the temporal evolution of the degree distribution, as shown by the cores of the boxplots (interquartile range) overlapping.

In Figure S11 we show the duration of each DWM in our dataset, color-coding by the average daily volume of transactions in USD. Our dataset covers all major DWMs from their onstart in 2011 with Silk Road Marketplace. DWMs are heterogeneous in daily volume, with some being just over our threshold of 20,000 USD, and Hydra Marketplace or AlphaBay Market close to 1M daily USD.

In Figure S12 we show the size of the 144 product markets in the regulated e-commerce platform dataset. In Figure S12(a), we show the total number of transactions in each product market, whereas in Figure S12(b) we show the total number of users (buyers and sellers). Product markets are heterogeneous in size, both w.r.t. number of transactions and number of users.

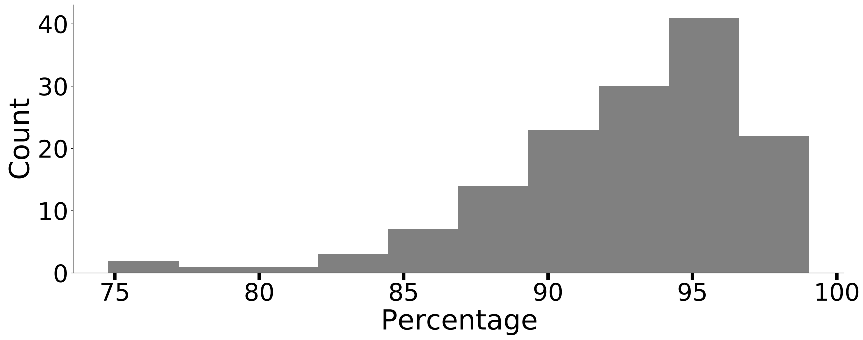

Figure S1: **Most buyers with zero entropy only have done one transaction.** Histogram of percentage of buyers doing just one transaction for each e-commerce product market, among those with zero entropy. In most markets the percentage is well above 90%.

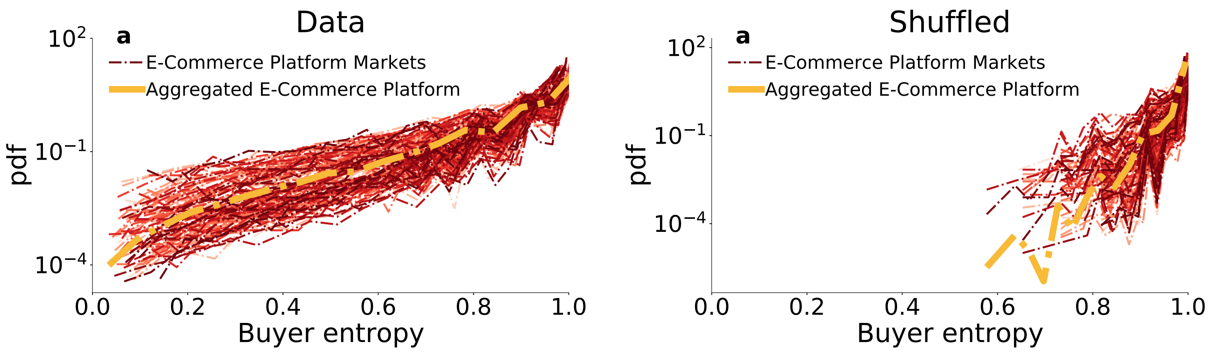

Figure S2: **The entropy distribution is significant against a null model.** (a) Buyer entropy distribution for each market (red) and all markets (yellow) in the e-commerce datasets. (b) Same distributions build reshuffling the transaction links, such that buyers have the same number of transactions, but with random sellers. The distributions with the reshuffled data show considerably higher entropy, meaning that the buyers in the dataset show non-random effects of memory.

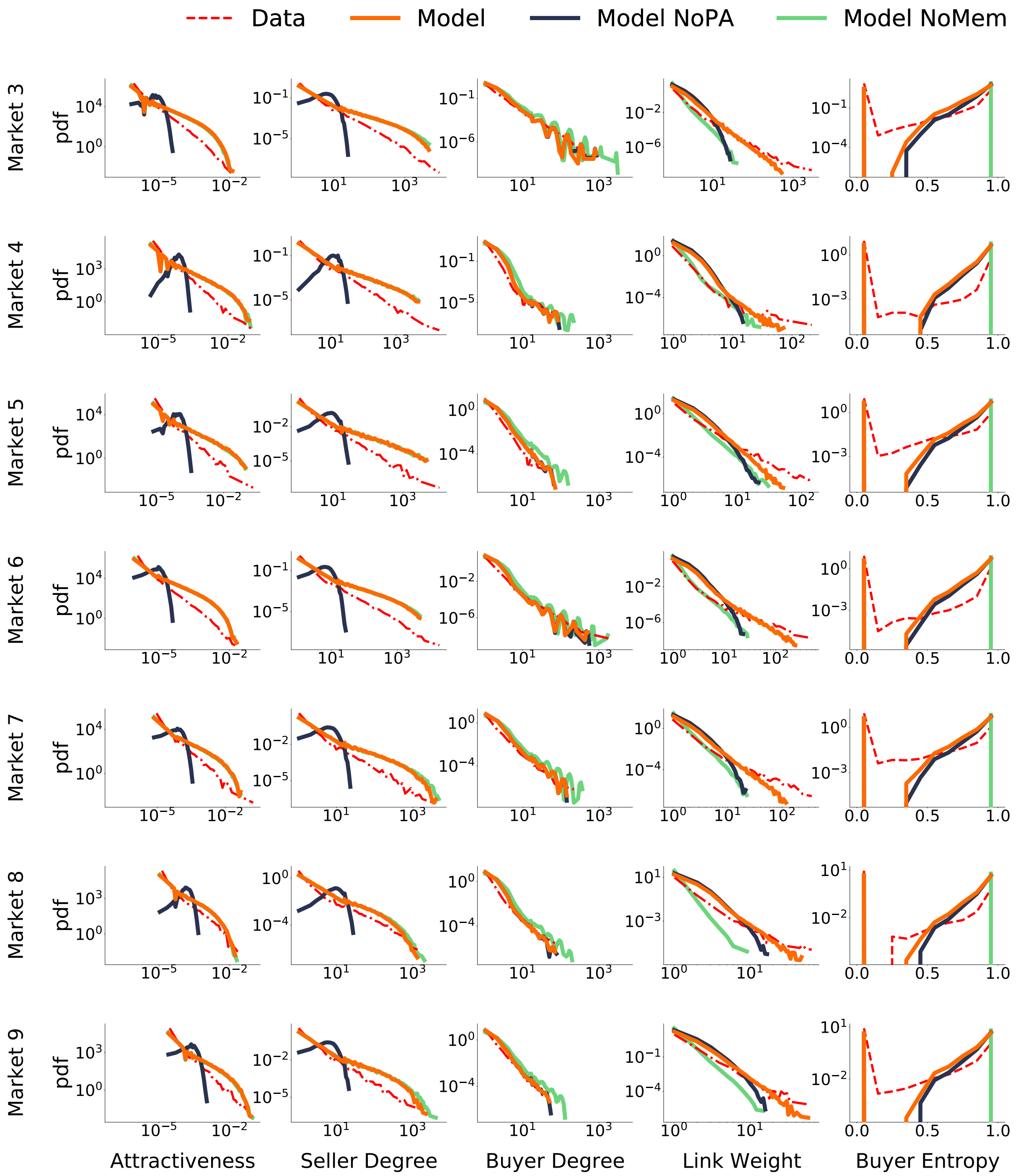

Figure S3: **Model simulations for different markets - final distributions - Markets 3 to 9.** Each row corresponds to a different market, whose simulations parameters are individually calibrated as detailed in the main text. From left to right, we show distributions for different quantities: attractiveness, seller degree, buyer degree, link weight and seller entropy. The comparison with the two model variations, without preferential attachment or without memory, shows the key role of both parameters in shaping the network: preferential attachment is crucial in reproducing highly active sellers, whereas buyer memory is fundamental to capture the heterogeneity of buyer-seller relationships.

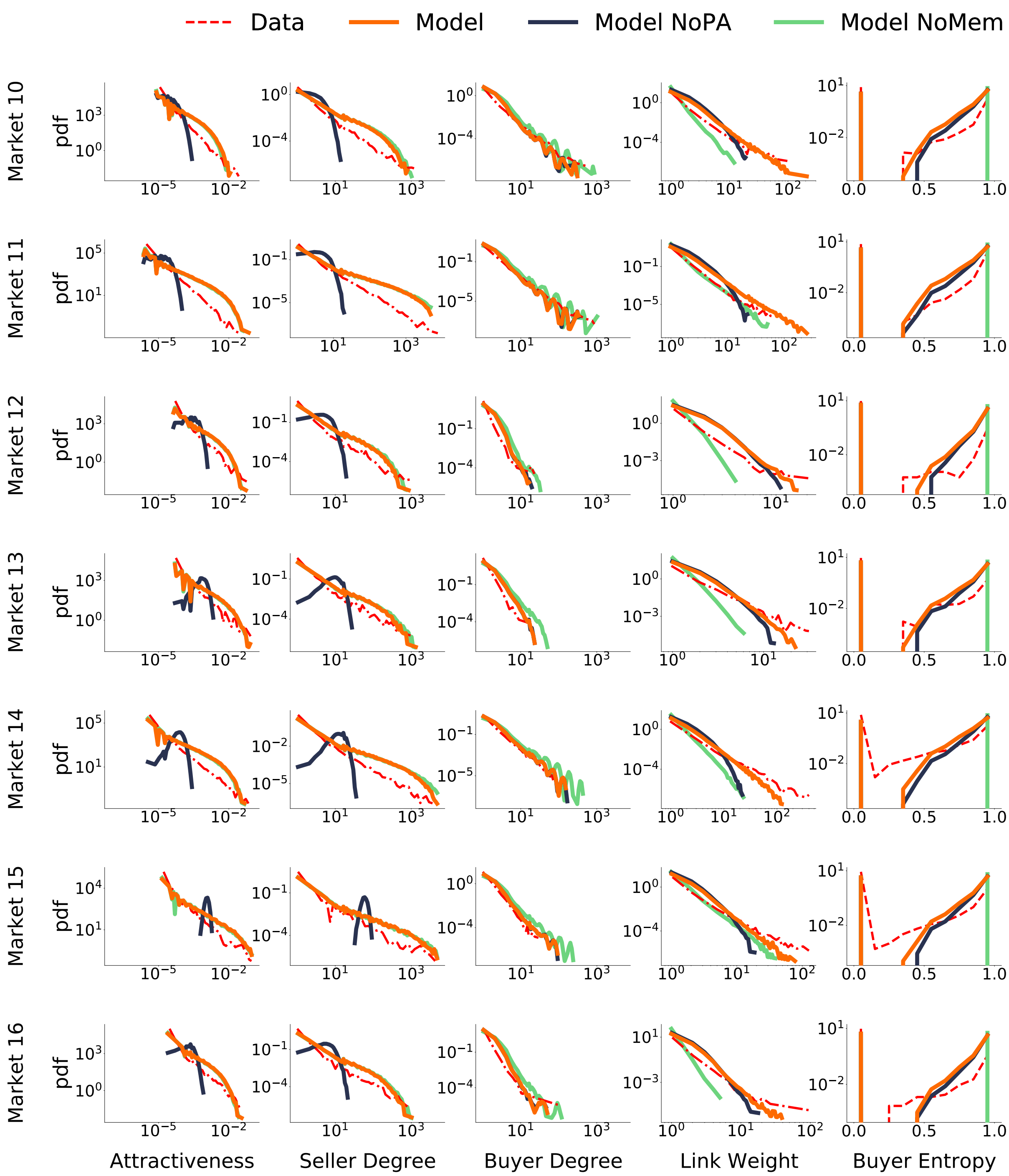

Figure S4: **Model simulations for different markets - final distributions - Markets 10 to 16.** Each row corresponds to a different market, whose simulations parameters are individually calibrated as detailed in the main text. From left to right, we show distributions for different quantities: attractiveness, seller degree, buyer degree, link weight and seller entropy. The comparison with the two model variations, without preferential attachment or without memory, shows the key role of both parameters in shaping the network: preferential attachment is crucial in reproducing highly active sellers, whereas buyer memory is fundamental to capture the heterogeneity of buyer-seller relationships.

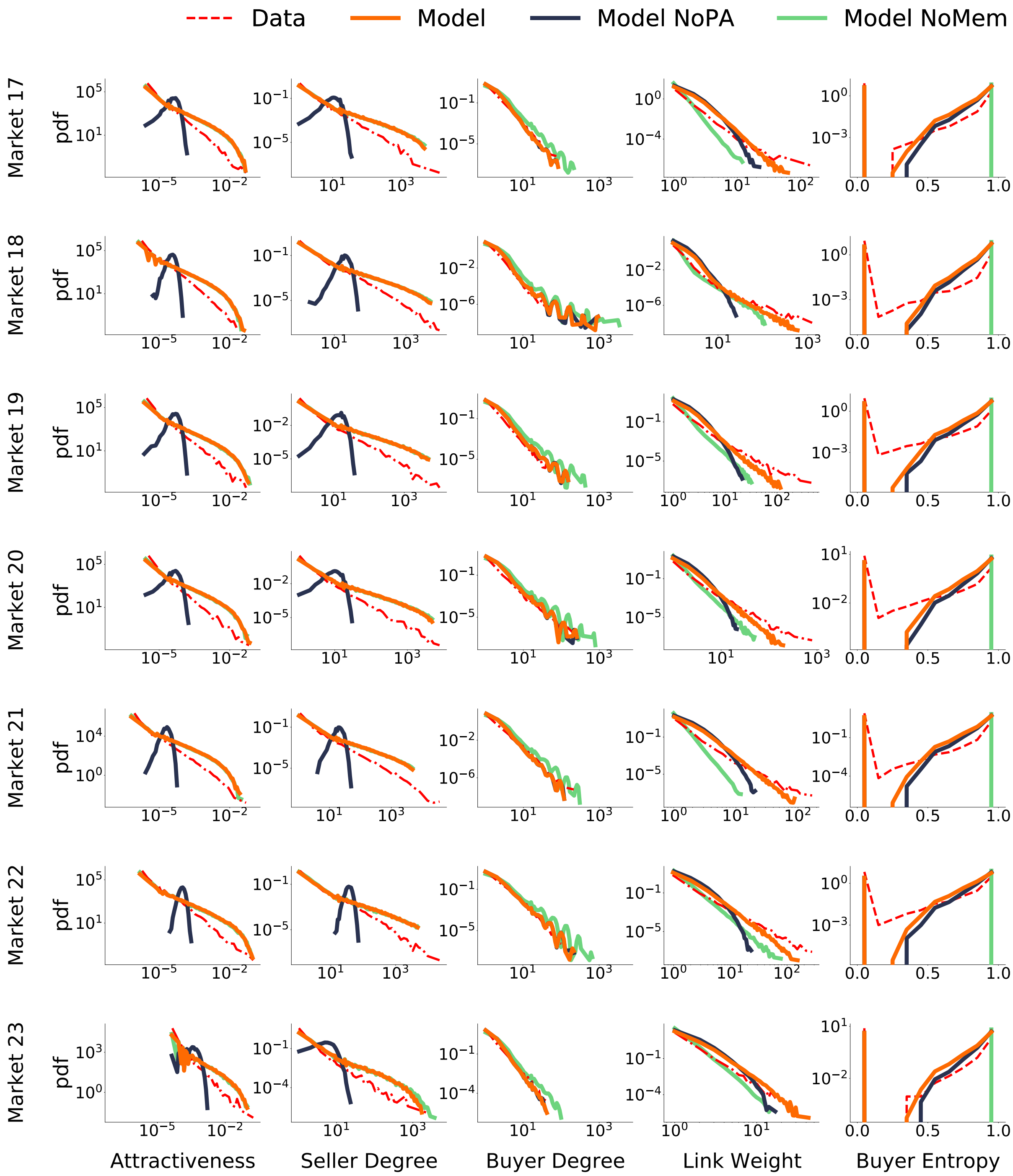

Figure S5: **Model simulations for different markets - final distributions - Markets 17 to 23** Each row corresponds to a different market, whose simulations parameters are individually calibrated as detailed in the main text. From left to right, we show distributions for different quantities: attractiveness, seller degree, buyer degree, link weight and seller entropy. The comparison with the two model variations, without preferential attachment or without memory, shows the key role of both parameters in shaping the network: preferential attachment is crucial in reproducing highly active sellers, whereas buyer memory is fundamental to capture the heterogeneity of buyer-seller relationships.

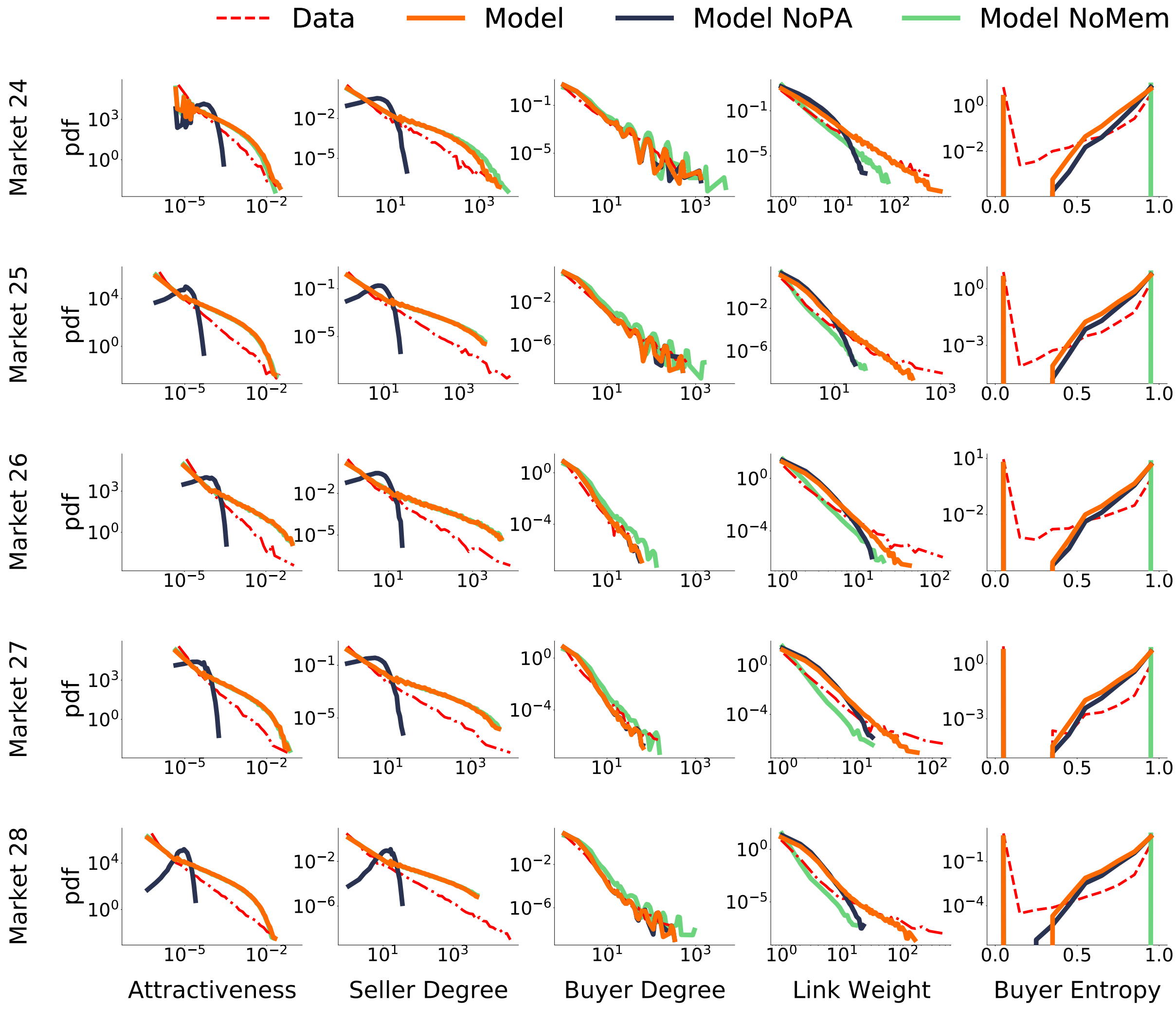

Figure S6: **Model simulations for different markets - final distributions - Markets 24 to 28** Each row corresponds to a different market, whose simulations parameters are individually calibrated as detailed in the main text. From left to right, we show distributions for different quantities: attractiveness, seller degree, buyer degree, link weight and seller entropy. The comparison with the two model variations, without preferential attachment or without memory, shows the key role of both parameters in shaping the network: preferential attachment is crucial in reproducing highly active sellers, whereas buyer memory is fundamental to capture the heterogeneity of buyer-seller relationships.

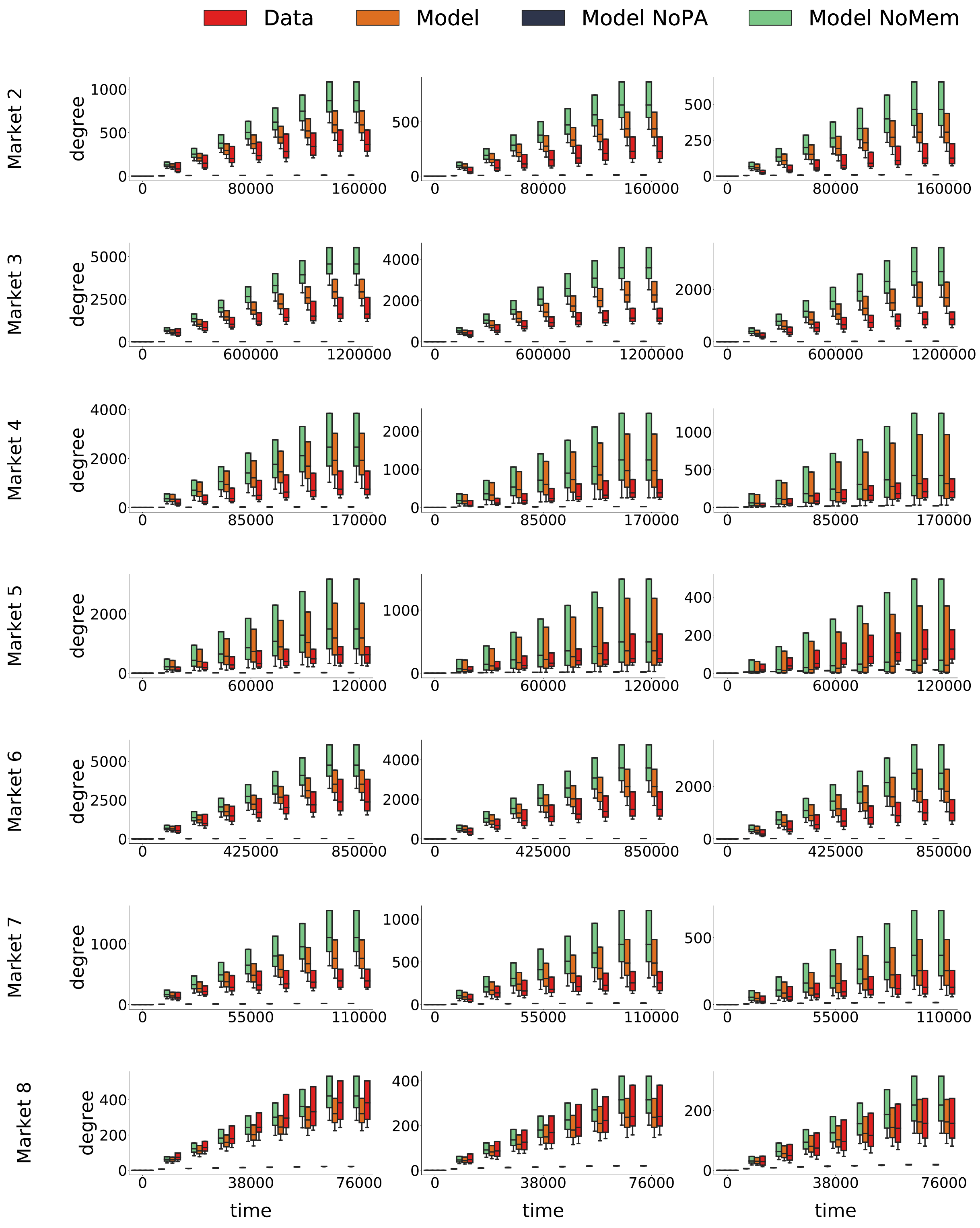

Figure S7: **Model simulations for different markets - temporal evolution - Markets 2 to 8** Each row represents one market. From left to right: temporal evolution of the degree distribution of the top 50 (left), 100 (center) and 200(right) sellers, representing the distribution at 9 equally spaced time steps with boxplots ranging from the first to the third quartiles, whiskers extending from 2.5^{th} to 97.5^{th} percentiles. The model better captures the temporal evolution of the top sellers degree for all product markets than the alternatives neglecting either the preferential attachment or the memory mechanism.

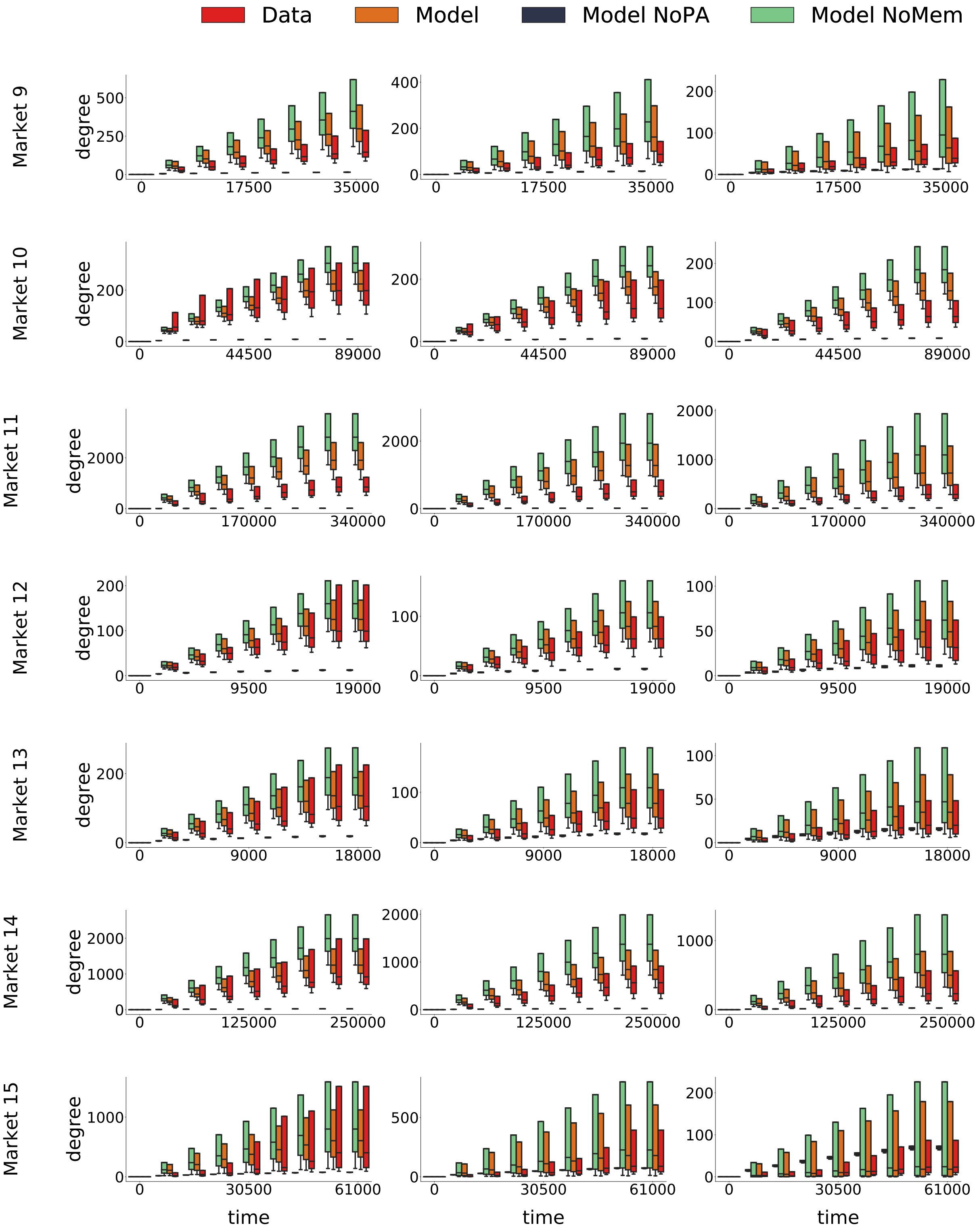

Figure S8: **Model simulations for different markets - temporal evolution - Markets 9 to 15** Each row represents one market. From left to right: temporal evolution of the degree distribution of the top 50 (left), 100 (center) and 200(right) sellers, representing the distribution at 9 equally spaced time steps with boxplots ranging from the first to the third quartiles, whiskers extending from 2.5^{th} to 97.5^{th} percentiles. The model better captures the temporal evolution of the top sellers degree for all product markets than the alternatives neglecting either the preferential attachment or the memory mechanism.

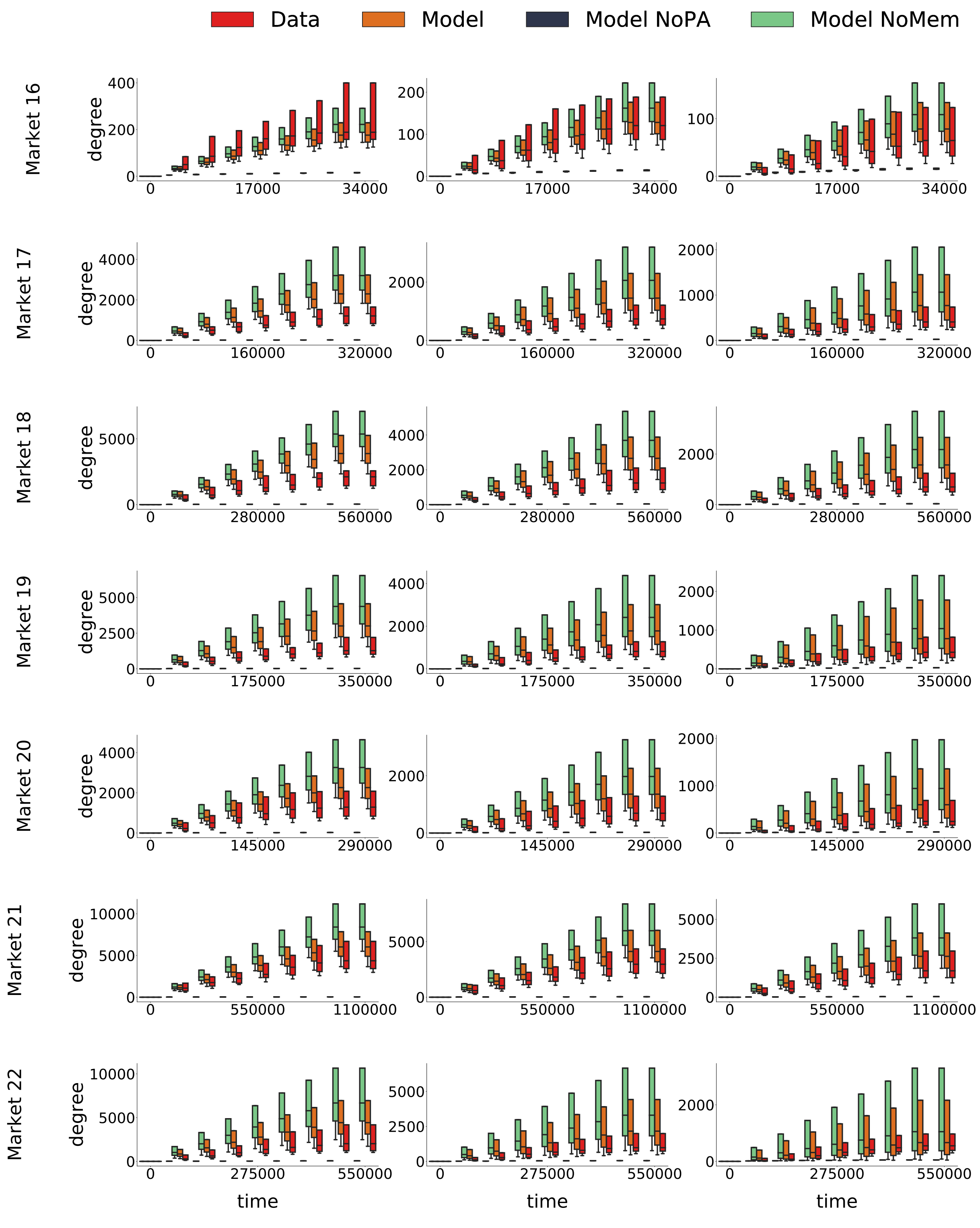

Figure S9: **Model simulations for different markets - temporal evolution - Markets 16 to 22** Each row represents one market. From left to right: temporal evolution of the degree distribution of the top 50 (left), 100 (center) and 200(right) sellers, representing the distribution at 9 equally spaced time steps with boxplots ranging from the first to the third quartiles, whiskers extending from 2.5^{th} to 97.5^{th} percentiles. The model better captures the temporal evolution of the top sellers degree for all product markets than the alternatives neglecting either the preferential attachment or the memory mechanism.

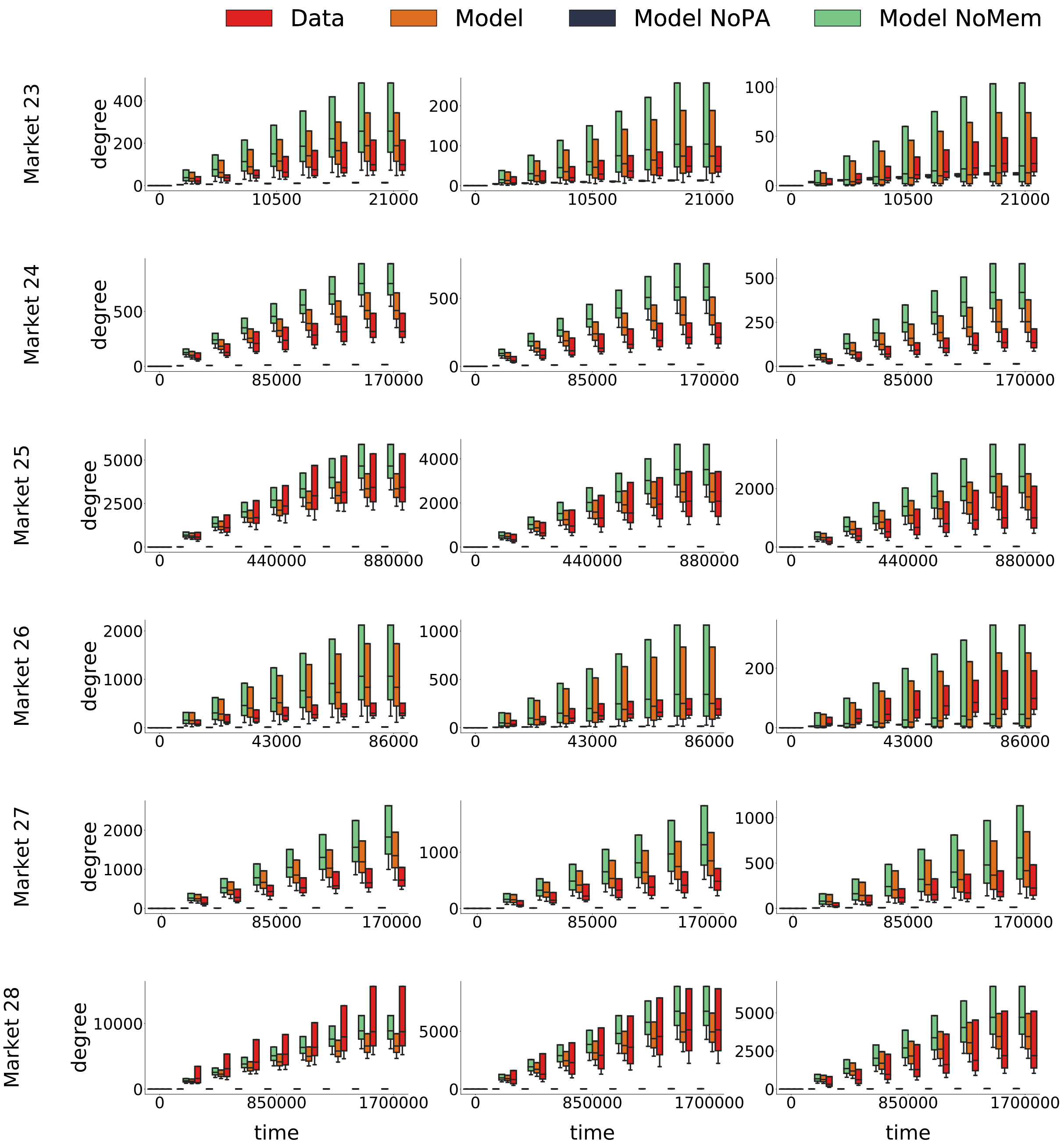

Figure S10: **Model simulations for different markets - temporal evolution - Markets 23 to 28** Each row represents one market. From left to right: temporal evolution of the degree distribution of the top 50 (left), 100 (center) and 200(right) sellers, representing the distribution at 9 equally spaced time steps with boxplots ranging from the first to the third quartiles, whiskers extending from 2.5^{th} to 97.5^{th} percentiles. The model better captures the temporal evolution of the top sellers degree for all product markets than the alternatives neglecting either the preferential attachment or the memory mechanism.

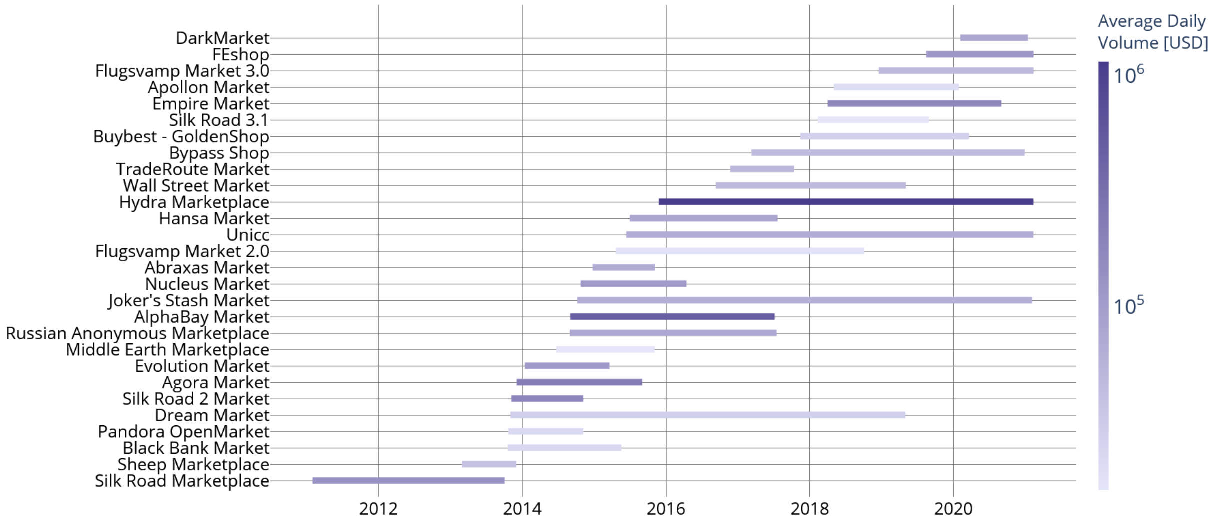

Figure S11: **Dark Web Marketplaces** Duration of each market in our dataset, color coded by the number of transactions involving each marketplace. Each market is live at least for 180 days and averages at least 20'000 transactions per day.

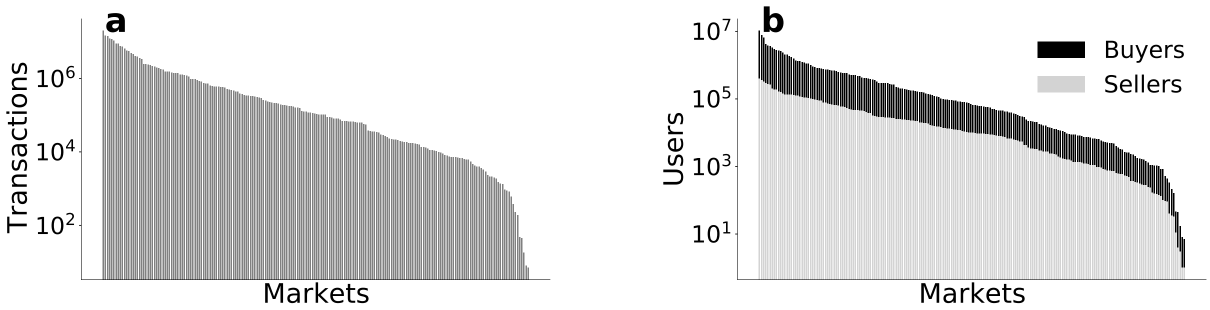

Figure S12: **E-Commerce Platform Markets** a): Plot of the number of transaction per each market of the e-commerce platform data. b): number of buyers and sellers in each market of the e-commerce platform data.

Table S1: Memory kernel fitted coefficients β and c for every degree class k_{min} <degree< $k_{min} * 2$

k_{min}	β	c
2	0.0663 ± 0.0003	0.00100 ± 0.00004
4	0.0561 ± 0.0003	0.00100 ± 0.00004
8	0.0717 ± 0.0003	0.0051 ± 0.0001
16	0.0914 ± 0.0004	0.0124 ± 0.0003
32	0.1026 ± 0.0004	0.0166 ± 0.0005
64	0.1010 ± 0.0006	0.0115 ± 0.0005
128	0.0927 ± 0.0008	0.0058 ± 0.0005

Supplementary Tables

In Table S1 we show the fitted coefficients of β and c for every buyer degree class. The coefficients are slightly different among the different degree classes, but of the same order of magnitude, allowing us to fix the value of $beta$ and c in the model simulation. This is only an approximation in the context of a model whose goal is to see the role of different mechanisms in determining the structure and evolution of the buyer-seller network. If the goal was to reproduce the finest detail of the network, or to make predictions on its evolution, we'd assign values of β and c according to the buyer degree class, where the degree is sampled from the data degree distribution.

In Table S2 we show details on each DWM in the dataset. Details include the name, start and end data, reason of closure, type of goods traded, total number of transactions and total volume of transactions in USD.

In Table S3 we show the value of the preferential attachment parameter μ fitted for each product market. While values are heterogeneous, showing the different role of preferential attachment in each market, the precise value is not important in our study. Indeed, our only goal is to reproduce the main stylized facts of the data, not to reproduce the finest details of the network, and therefore changing the value of μ around the fitted value would not change our conclusions.

Table S2: Details of the DWMs under study: start, end, reason of closure, type, total number of transactions and total volume

Name	Start	End	Closure	Type	#Trx	Volume [USD]
Silk Road Marketplace	2011-01-31	2013-10-02	raided	mixed	840,987	131,604,274
Sheep Marketplace	2013-02-28	2013-11-29	scam	drugs	65,904	10,923,327
Black Bank Market	2013-10-18	2015-05-18	scam	mixed	89,444	13,152,830
Pandora OpenMarket	2013-10-20	2014-11-05	raided	drugs	73,127	8,401,191
Dream Market	2013-11-01	2019-04-30	voluntary	mixed	570,734	57,637,323
Silk Road 2 Market	2013-11-06	2014-11-05	raided	mixed	426,277	66,825,593
Agora Market	2013-12-03	2015-09-01	voluntary	mixed	911,094	141,473,388
Evolution Market	2014-01-14	2015-03-19	scam	drugs	372,822	47,578,872
Middle Earth Marketplace	2014-06-22	2015-11-04	scam	mixed	67,630	8,361,143
Russian Anonymous Marketplace	2014-08-29	2017-07-15	raided	mixed	1,109,126	80,478,841
AlphaBay Market	2014-09-01	2017-07-05	raided	mixed	4,263,740	546,010,808
Joker's Stash Market	2014-10-07	2021-02-03	closed	credits	998,687	153,138,403
Nucleus Market	2014-10-24	2016-04-13	scam	mixed	391,394	56,594,214
Abraxas Market	2014-12-24	2015-11-05	scam	drugs	168,642	21,854,042
Flugsvamp Market 2.0	2015-04-20	2018-10-02	closed	drugs	254,972	23,013,741
Unicc	2015-06-13	2021-02-10	active	credits	2,930,842	147,814,198
Hansa Market	2015-07-01	2017-07-20	raided	drugs	617,414	60,644,436
Hydra Marketplace	2015-11-25	2021-02-10	active	mixed	6,005,608	2,175,558,739
Wall Street Market	2016-09-09	2019-05-03	raided	mixed	681,825	48,153,667
TradeRoute Market	2016-11-22	2017-10-12	scam	mixed	137,722	16,969,504
Bypass Shop	2017-03-10	2020-12-27	closed	unknown	1,041,438	65,663,561
Buybest - GoldenShop	2017-11-13	2020-03-19	closed	unknown	386,046	24,449,110
Silk Road 3.1	2018-02-10	2019-08-27	scam	drugs	93,426	9,053,684
Empire Market	2018-04-01	2020-08-30	scam	mixed	454,473	154,457,692
Apollon Market	2018-05-03	2020-01-27	scam	drugs	106,395	12,902,953
Flugsvamp Market 3.0	2018-12-17	2021-02-10	active	unknown	291,018	39,344,294
FEshop	2019-08-14	2021-02-10	active	unknown	1,342,574	64,666,841
DarkMarket	2020-02-04	2021-01-12	raided	unknown	363,825	27,246,084

Table S3: **Preferential attachment parameter μ.** Values of the preferential attachment parameter μ for each product market, fitted with maximum likelihood estimation on the attractiveness distribution.

Market	μ
1	85
2	21
3	155
4	225
5	400
6	220
7	90
8	14
9	50
10	27
11	290
12	15
13	13
14	70
15	33
16	14
17	175
18	140
19	240
20	210
21	180
22	200
23	65
24	35
25	190
26	410
27	220
28	230

References

[1] Roger Dingledine, Nick Mathewson, and Paul Syverson. Tor: The second-generation onion router. In *Proceedings of the 13th Conference on USENIX Security Symposium - Volume 13*, SSYM'04, page 21, USA, 2004. USENIX Association.

[2] Darknetlive, 2020.

[3] Danny Yuxing Huang, Maxwell Matthaios Aliapoulios, Vector Guo Li, Luca Invernizzi, Elie Bursztein, Kylie McRoberts, Jonathan Levin, Kirill Levchenko, Alex C Snoeren, and Damon McCoy. Tracking ransomware end-to-end. In *2018 IEEE Symposium on Security and Privacy (SP)*, pages 618–631. IEEE, 2018.

[4] Mikkel Alexander Harlev, H. Yin, Klaus Christian Langenheldt, R. Mukkamala, and R. Vatrapu. Breaking Bad: De-Anonymising Entity Types on the Bitcoin Blockchain Using Supervised Machine Learning. In *HICSS*, 2018.

[5] Daniel Goldsmith, Kim Grauer, and Yonah Shmalo. Analyzing hack subnetworks in the bitcoin transaction graph. *Applied Network Science*, 5:1–20, 2020.

[6] Paolo Tasca, Adam Hayes, and Shaowen Liu. The evolution of the bitcoin economy: Extracting and analyzing the network of payment relationships. *The Journal of Risk Finance*, 19(2):94–126, March 2018.

[7] Dorit Ron and Adi Shamir. Quantitative analysis of the full bitcoin transaction graph. In *International Conference on Financial Cryptography and Data Security*, pages 6–24. Springer, 2013.

[8] Sarah Meiklejohn, Marjori Pomarole, Grant Jordan, Kirill Levchenko, Damon McCoy, Geoffrey M Voelker, and Stefan Savage. A fistful of bitcoins: Characterizing payments among men with no names. In *Proceedings of the 2013 Conference on Internet Measurement Conference*, pages 127–140, 2013.

[9] Dmitry Ermilov, Maxim Panov, and Yury Yanovich. Automatic bitcoin address clustering. In *2017 16th IEEE International Conference on Machine Learning and Applications (ICMLA)*, pages 461–466. IEEE, 2017.

[10] Elli Androulaki, Ghassan O Karame, Marc Roeschlin, Tobias Scherer, and Srdjan Capkun. Evaluating user privacy in bitcoin. In *International Conference on Financial Cryptography and Data Security*, pages 34–51. Springer, 2013.

[11] Lily Hay Newman. How a Bitcoin Trail Led to a Massive Dark Web Child-Porn Site Takedown, 2019.

[12] Gwern Branwen, Nicolas Christin, David Décary-Hétu, Rasmus Munksgaard Andersen, StExo, El Presidente, Anonymous, Daryl Lau, Delyan Kratunov Sohhlz, Vince Cakic, Van Buskirk, Whom, Michael McKenna, and Sigi Goode. Dark net market archives, 2011-2015, July 2015.
